# Fat Mass and Obesity Associated Gene (*FTO*) Expression Is Regulated Negatively by the Transcription Factor Foxa2

**DOI:** 10.1371/journal.pone.0051082

**Published:** 2012-12-07

**Authors:** Jianjin Guo, Wei Ren, Ying Ding, Aimei Li, Lu Jia, Dongming Su, Xiang Liu, Kuanfeng Xu, Tao Yang

**Affiliations:** 1 Department of Endocrinology, The First Affiliated Hospital of Nanjing Medical University, Nanjing, Jiangsu Province, China; 2 Department of Endocrinology and Metabolism, Shanghai Jiaotong University Affiliated Sixth People’s Hospital; Shanghai Diabetes Institute; and Shanghai Clinical Center of Diabetes, Shanghai, China; 3 The Center of Metabolic Disease Research, Nanjing Medical University, Nanjing, Jiangsu Province, China; 4 Department of Microbiology and Immunology, Medical University of South Carolina, Charleston, South Carolina, United States of America; Pennington Biomedical Research Center, United States of America

## Abstract

Fat mass and obesity associated gene (*FTO*) is the first gene associated with body mass index (BMI) and risk for diabetes. *FTO* is highly expressed in the brain and pancreas, and is involved in regulating dietary intake and energy expenditure. To investigate the transcriptional regulation of *FTO* expression, we created 5′-deletion constructs of the *FTO* promoter to determine which transcription factors are most relevant to *FTO* expression. The presence of an activation region at −201/+34 was confirmed by luciferase activity analysis. A potential Foxa2 (called HNF-3β) binding site and an upstream stimulatory factor (USF)-binding site was identified in the −100 bp fragment upstream of the transcription start site (TSS). Furthermore, using mutagenesis, we identified the Foxa2 binding sequence (−26/−14) as a negative regulatory element to the activity of the human *FTO* promoter. The USF binding site did not affect the *FTO* promoter activity. Chromatin immunoprecipitation (ChIP) assays were performed to confirm Foxa2 binding to the *FTO* promoter. Overexpression of Foxa2 in HEK 293 cells significantly down-regulated *FTO* promoter activity and expression. Conversely, knockdown of Foxa2 by siRNA significantly up-regulated FTO expression. These findings suggest that Foxa2 negatively regulates the basal transcription and expression of the human *FTO* gene.

## Introduction

Overweight and obesity are independent risk factors for type 2 diabetes, cancer, and coronary artery disease, posing serious threats to public health [1]–[3]. In 2007, fat mass and obesity-associated gene (*FTO*) was discovered in a genome-wide association study (GWAS) for obesity (or obesity-related traits) [4]. During the past few years, research has focused on identifying the relationship between SNP genotypes and obesity [5]–[6]. For instance, a meta-analysis investigated the associations between five *FTO* polymorphisms (rs9939609, rs1421085, rs8050136, rs17817449, and rs1121980) and obesity risk in 41,734 cases and 69,837 controls from 59 studies and concluded that the SNPs account for the risk of obesity to various degrees [7]. For example, a significant association between obesity and rs9939609 was detected, with an overall OR of 1.31 (95% CI: 1.26 to 1.36). Growing evidence suggests that some *FTO* polymorphisms are closely related to the risk and outcome of insulin resistance and diabetes [8]–[10]. If one ignores the effect of BMI, the association between SNP rs9939609 and type 2 diabetes is abolished, suggesting that the association is causally related to increased BMI [11]. Several reports have found that the *FTO* SNPs are significantly associated with breast cancer and endometrial cancer as well [2], [12]–[13]. FTO is highly expressed in the hypothalamus and pancreatic islets, and widely expressed at a lower level in multiple tissues including adipose tissue, liver, and skeletal muscle. Several types of stimuli could alter the FTO expression level. For instance, food deprivation results in the down-regulation of FTO expression in the mouse hypothalamus [14]–[15] but up-regulation in the rat hypothalamus [16]. In contrast, over-expression of FTO in mice increases food intake, leading to obesity. Furthermore, Bravard et al. observed a significant increase of *FTO* mRNA and protein levels in muscle tissue of patients with type 2 diabetes, resulting in altered insulin signaling, and increased lipogenesis and reactive oxygen species levels (ROS). Other data support a potential role for FTO in oxidative metabolism, lipogenesis and oxidative stress in muscle [16]. Moreover, induced expression of FTO leads to enhancement of the first phase of glucose-induced insulin secretion in INS-1 cells. However, the molecular mechanisms controlling the expression of FTO are not completely understood.

Understanding the transcriptional regulation of *FTO* expression will be important in elucidating the role of FTO in the regulation of energy balance. It may also point to new therapeutic strategies for inhibiting or activating FTO activity in patients with obesity or diabetes. In order to explore the mechanisms of its transcriptional regulation, we identified and characterized the promoter of the human *FTO* gene. By generating a series of 5′ deletions, we revealed that the core promoter is located within -201 relative to the transcription start site (TSS). Using the promoter screening method, three Sp1, one Foxa2 and a USF binding site were selected for further study. Luciferase reporter studies were carried out to investigate the functions of these factors in regulating *FTO* promoter activity in Hela and HEK 293 cells. We found that two Sp1 sites in the region (-201/101) can strongly transactivate the *FTO* promoter. Surprisingly, results from fluorescence measurements showed that mutation of the Foxa2 binding site increased the promoter activity of *FTO*, while the USF binding site did not affect the *FTO* promoter activity. Electrophoretic mobility shift assay (EMSA) and Chromatin immunoprecipitation (ChIP) analysis demonstrated that Foxa2 associates with the binding sites of the *FTO* gene *in vitro* and *in vivo*. Overexpression of Foxa2 decreased the level of FTO, whereas knockdown of Foxa2 by siRNA led to increased FTO expression. These results suggest that the Foxa2 transcription factor binding sequence AGCCTGTTTGCTC (−26/−14) is the negative regulatory element of *FTO*.

The Foxa2 transcription factor is essential for glucose and lipid homeostasis, especially for insulin secretion from β cells [17]–[18]. A recent study has revealed the surprising fact that Foxa2 binds to the melanin-concentrating hormone (MCH) and orexin promoters and regulates their expression in the lateral hypothalamic area, where *FTO* gene expression is frequently detected [19]. Our study shows that Foxa2 binds to the promoter and inhibits the expression of *FTO*. The transcription factor Foxa2 may play a vital role in regulating the balance of food intake and other biological actions.

## Materials and Methods

### Cell Lines and Plasmids

The human embryonic kidney cell line HEK 293 and the human cervical carcinoma cell line Hela were obtained from Invitrogen and maintained in DMEM (Invitrogen) supplemented with 10% fetal bovine serum and 1% antibiotic-antimycotic agents.

PCR was used to generate 5′ stepwise deletion constructs of the *FTO* promoter. An approximately 2.0 kb *KpnI/BglII* fragment (−2028 to +34) which contains the 5′-flanking region of the *FTO* gene was isolated by PCR amplification from HEK 293 cells DNA, and then ligated into the *KpnI/BglII*-digested pGL3-Basic vector (Promega). The resulting vector allows for the monitoring of the level of gene expression by the firefly luciferase gene under the control of this promoter fragment. The resulting plasmid (pGL3-2k) was used as a template to synthesize a series of deletion reporter gene constructs. PCR was performed using sets of oligonucleotide primers specific for the human *FTO* gene sequence, of which the forward primer was the *KpnI*-site-linked, and the reverse primer was a *BglII*-site-linked (Table 1). All plasmid DNAs were purified using the QIAfilter plasmid kit (Qiagen) and then sequenced to confirm to the sequence of the insert (Takara). The expression plasmid encoding the human Foxa2 (pHD-Foxa2) and the control plasmid were kindly provided by Dr. Klaus H. Kaestner (Department of Genetics, University of Pennsylvania School of Medicine).

**Table 1:** The primer sequences for the preparation of promoter-reporter plasmids.

Names of plasmids	Primer sequence(5′-3′): sense+antisense
PGL3-2028	F1: CGG**GGTACC**AATCCTTGGGAGATCATCTACTTR1: GGA**AGATCT**GAATTTCCCAGGTCCGT
PGL3-1390	F2: CGG**GGTACC**AGCTGGTTCCTTAATCTTTGG+R1
PGL3-1072	F3: CGG**GGTACC**TAAACATCTTGAGACGGGTAT+R1
PGL3-882	F4: CGG**GGTACC**CAACATGATGAAACCCTGTCT+R1
PGL3-716	F5: CGG**GGTACC**CAACGGAGCAAGAACCTGTCA+R1
PGL3-340	F6: CGG**GGTACC**GGCCTGAGGATGTGGAGGTGTC+R1
PGL3-201	F7: CGG**GGTACC**TCCACCCACCCTCATCCTCC+R1
PGL3-100	F8: CGG**GGTACC**TGGCCGAGAGGAGCACGGGA+R1

Restriction endonuclease sequences are shown in bold.

### Transient Transfections and Luciferase Assay

For assaying luciferase expression, HEK 293 and Hela cells (5 to 7.5×10^4^) were seeded onto 24-well plates, cultured overnight, and cotransfected with 800 ng of the pGL3 vector reporter construct and 200 ng of pRL-TK (Promega) as a transfection efficiency control. For all experiments, cells were cultured for 24 h after transfection, and cell lysates were measured using the Dual Luciferase Reporter Assay System Kit (Promega). Luminescence was determined in a Modulus luminometer (Turner Biosystems) after addition of substrate to allow adequate mixing. Relative firefly luciferase activities (RLU) were calculated by normalizing transfection efficiencies with the Renilla luciferase activity. All the data shown in this study were obtained from at least three independent experiments.

### Bioinformatics Transcriptional Elements Analyses

To identify transcriptional regulatory sequences and potential transcription factor binding sites on the putative promoter regions. We analyzed the 5′-flanking region from 240 bp upstream of the transcription start site with TFsearch (http://mbs.cbrc.jp/research/db/TFSEARCH.html) and AliBaba 2.1 (http://www.gene-regulation.com).

### Site-directed-mutagenesis

Substitution mutation constructs of USF and Foxa2 binding sites in pGL3-100 were generated using a MutanBEST site-directed mutagenesis kit (Takara) with the pGL3-100 plasmid as the template. The mutagenesis primers designed for the mutations were as follows (the mutated sequences are underlined): mu-USF-Forward: 5′-GAAACATGGCAGGCTCCCGT-3′, Reverse: 5′-TCCCAGTCTCCTCTCGGCCA-3′; mu-Foxa2-Forward: 5′-GACTCTAGCCGACTTGCTCGCG-3′, Reverse: 5′-CAAG GAGAACTACATTTCC-3′. The mu-USF/Foxa2 was created using mu-Foxa2 as template. In the mutant expression clones, the sequences of the entire region mutated were amplified by PCR and the expected mutations were verified by DNA sequencing.

### Electrophoretic Mobility Shift Assay (EMSA)

For the gel shift assay, double-strand DNA oligonucleotide probes were synthesized with a biotin label at the 5′ end (Invitrogen, Shanghai, China). The probes used included the following double-stranded oligonucleotides: Foxa2 wild type 5′-CTCTAG CCTGTTTGCTCGCG-3′; Foxa2 mutant 5′-CTCTAGCCGACCGGCTCGCG-3′ (mutated bases shown as underline). Nuclear extracts were prepared from cultured HEK 293 cells using NE-PER nuclear and cytoplasmic extraction reagent kit (Pierce, Rockford, IL, USA). The protein concentrations were determined using the Bradford protein assay kit (Pierce, USA) following the manufacturer’s protocol. EMSA was conducted using a Light Shift Chemiluminescent EMSA kit (Pierce, USA) as recommended by the manufacturer’s instructions.

Nuclear extracts containing 15 ug of protein were incubated with the poly[d(I-C)], the binding buffer and oligonucleotide probes for 20 minutes at 25°C. In the competition experiments, mutant probe or 100-fold excess of cold oligonucleotides were pre-incubated with the nuclear extracts for 10 minutes before the labeled probes were added to the reaction. The antibody for Foxa2 (Santa Cruz, CA, USA) were used to carry out super-EMSA.

### Chromatin Immunoprecipitation Assays

ChIP assays were performed according to the manufacturer’s instructions (Active Motif, Carlsbad, CA). The DNA/protein complex were immunoprecipitated using a Foxa2 antibody (Santa Cruz) and the DNA was purified using gel exclusion columns. The purified ChIP DNA fragment was subjected to semiquantitative PCR analysis (1 cycle of 95°C for 3 min, 35 cycles of 95°C for 20 s, 64°C for 20 s, and 72°C for 1 min). Specific forward (5′-GCTAGCTACCGTTGCTATAGC-3′) and reverse (5′-CTGGAA GAGCGTAGTCCGCT-3′) primers were designed to amplify the *FTO* promoter region (−142 to +24 nucleotides in relation to the transcription start site). Amplification of the input chromatin before immunoprecipitation at a dilution of 1∶50 was used as a positive control. The PCR products were analyzed on a 2% agarose gel and quantified by densitometry using a Fluor’s fluorimeter and Quantity One software (Bio-rad).

### Overexpression and RNA Interference of Foxa2

In overexpression experiments, reporter plasmids were also cotransfected with pHD-Foxa2 expression vector or corresponding empty vector. In the RNA interference experiments, 50 nM siRNA specific to human Foxa2 or a control siRNA that does not target any sequence in the human genome (non-target control, NTC; Santa Cruz) were used in transient transfection. Total RNA was isolated 24 hour later and analyzed by RT-PCR. For western blotting experiments, lysates were obtained from cells cultured for 48 hours in 6-well plates.

### Real-time RT-PCR

Cells were transfected as described above for the luciferase assay. Total RNA was isolated according to the standard TRIZOL (Invitrogen) method. First-strand cDNA was synthesized from 1 µg of total RNA using M-MLV reverse transcriptase (Promega). Real time PCR was performed in ABI7500 (Applied Biosystems) using SYBR Green (Takara) using. Negative control reactions contained water instead of cDNA and were included in each run to ensure absence of contamination. Primers used were as follows: FTO-Forward, 5′-ACTTGGCTCCCTTATCTGACC and FTO-Reverse, 5′-TGTGCAGTGTGAGAAAGGCTT; GAPDH-Forward, 5′-AGGAC TCATGTCCATGCCAT-3′ and GAPDH-Reverse, 5′-ACCCTGTTGCTGTAGCCAAA [20]. Semi-quantitative RT-PCR was based on real-time quantitative reverse transcriptase polymerase chain reaction (qRT-PCR) results, so that the PCR reaction was stopped within the linear range of production. The amplified DNA fragments were visualized by agarose gel electrophoresis.

### Western Blot Analysis

Cells were harvested by scraping, washed in ice-cold PBS and lysed with 200 ul of lysis buffer containing a protease inhibitor cocktail (Roche). Protein concentrations were determined using the Bio-Rad Protein Assay (BioRad). Protein samples were prepared with 5×SDS sample buffer and loaded at 20 ug of protein per lane for SDS-PAGE. Western blot was performed with FTO (Abcam) and Foxa2 (Santa Sruz) antibodies, followed by goat anti-mouse IgG conjugated with HRP. GAPDH was detected as loading control. Chemoluminescence signals from three independent western analyses were quantified using an ECL imager, and analyzed using Quantity One software (BioRad).

### Statistical Analysis

Data are presented as means±SEM. Comparisons between means were performed by unpaired two-tailed Student’s *t*-test with Bonferroni correction as appropriate, using Microsoft Excel.

## Results

### The Core Promoter of Human *FTO* Gene is Located within −201 bp before TSS

To localize the active promoter regions and to determine the important transcription factors regulating human *FTO* gene expression, a nested series of 5′ truncated portions of the promoter were created extending up to 2.0 kb from the transcription start site of the human *FTO* gene. These constructs were transiently transfected in HEK 293 and Hela cells, and promoter activity was assessed by measuring luciferase activity.

**Figure 1:**
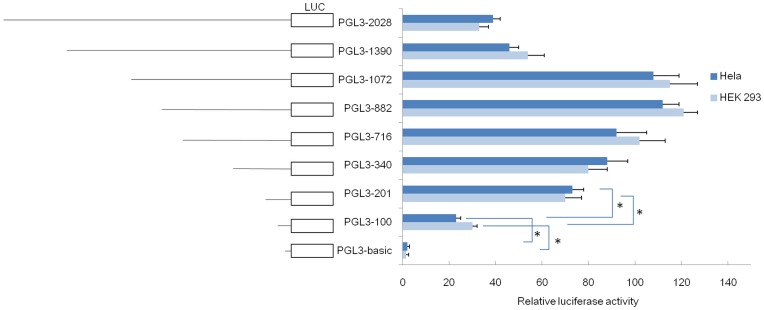
Functional analysis of the human *FTO* promoter. Different sizes of 5′deletion fragments of the human *FTO* promoter were cloned into pGL3-Basic luciferase reporter plasmids. The reporter construct was transfected into HEK 293 and Hela cell lines and the reporter activity was measured. Relative firefly luciferase activities were averages of three independent transfections normalized to renilla control activities. Data are presented a mean±S.D. (*P<0.05).

As shown in Fig. 1, deletions of the 5′-flanking region from nucleotide positions −2.0 kb (pGL3-2028) to −1.4 k (pGL3-1390) did not affect reporter gene activity. However, further deletion to the nucleotide position −1 k (pGL3-1072) resulted in a 2-fold increase in reporter activity, indicating that the region contains a negative regulatory element. No significant difference in luciferase activities among pGL3-1 k, pGL3-882, pGL3-716, pGL3-340 and pGL3-201 was observed. However, the luciferase activity of pGL3-100 was significantly less than that of pGL3-201. Moreover, pGL3-100 expressed a higher level of luciferase activity compared to the empty vector, suggesting that sequences in this region control the basal transcriptional activity of the *FTO* gene promoter which is located within the −201 bp before the transcription start site (TSS). Similar profiles of luciferase expression upon transfection with the reporter constructs were observed in both cell lines.

**Figure 2:**
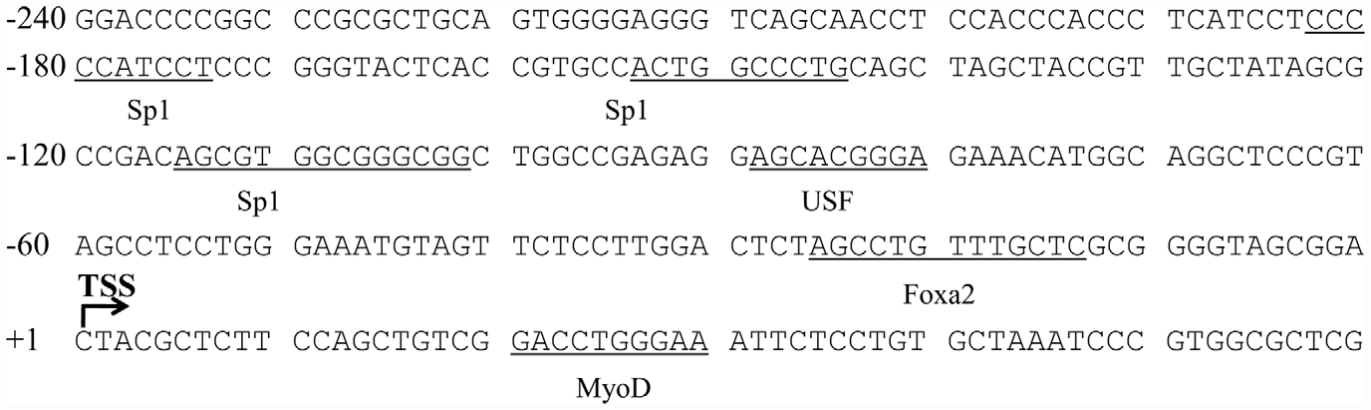
Putative binding sites in the *FTO* gene promoter region. The underlined sequences are putative binding sites for transcription factors in the *FTO* promoter region, based upon database searches using the TFsearch and Alibaba programs. The transcription start site (TSS) is indicated in the figure.

Based on the above findings, we analyzed the 5′-flanking region between 240 bp upstream to the transcription start site. Three Sp1 transcription factor binding sites are located between –201 and –100. A putative USF-binding site and a Foxa2 (HNF-3b) binding site were identified within the 100 bp before the TSS (Fig. 2).

### Foxa2 exerted a Dominant Negative Effect on the Activation of *FTO* Promoter

**Figure 3:**
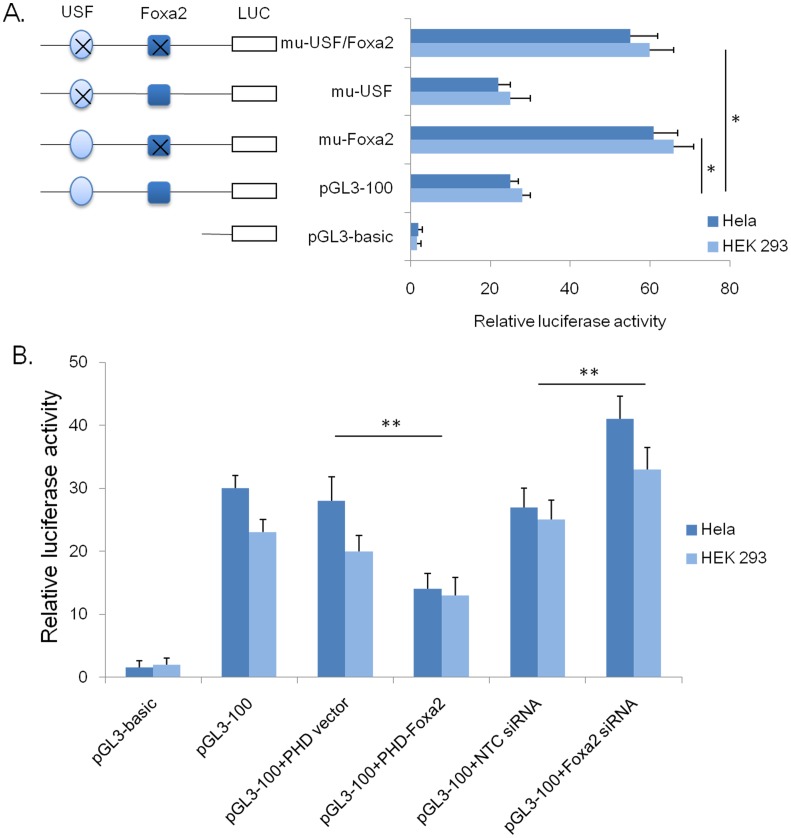
Characterization of the transcription factor binding elements by mutagenesis and luciferase assay. HEK 293 cells were transfected with the *FTO* promoter (PGL3-100), three mutant variants (mutated at the *Foxa2* site, USF site or both of them), pHD vector, pHD-Foxa2, negative control siRNA (NTC siRNA) or Foxa2 siRNA, and the promoter-less PGL3-basic construct. Relative luciferase activity (RLU), expressed as the fold induction relative to PGL3-basic vector, was measured. Results are presented as mean RLU±SE of three independent experiments (*P<0.05).

To investigate the role of the binding sites of transcription factors in the regulation of the *FTO* gene, mutants of the putative binding sites were constructed by site-directed mutagenesis. HEK 293 and Hela cells were transiently transfected with the mutant constructs. From these experiments we confirmed that the two Sp1 sites are essential for maintaining the basal transcriptional activity (data not shown). To further determine the function of the USF and Foxa2 binding sites, we measured the luciferase activities of the mutants pGL3-100/mu-USF, pGL3-100/mu-Foxa2 and pGL3-100/mu-USF/Foxa2 (Fig. 3A). It was observed that the luciferase activity of pGL3-100/mu-Foxa2 mutant was up-regulated by approximately 55% from that of pGL3-100 (*P*<0.01). In contrast, pGL3-100/mu-USF luciferase activity was not significantly different from pGL3-100. Furthermore, the level of luciferase activity of pGL3-100/mu-USF/Foxa2 matched that of pGL3-100/mu-Foxa2.

To explore the effect of Foxa2 on pGL3-100 luciferase activity, Foxa2 expression vector pHD-Foxa2 or a Foxa2 siRNA was transfected into HEK 293 and Hela cells (Fig. 3B). We determine that overexpression of dramatically reduced the relative luciferase activities (RLU) of pGL3-100 by 52% from that of the control vector groups. In addition, significantly increased the RLU in two cell lines transiently transfected with the Foxa2-siRNA over those treated with NTC siRNA. This observation suggests that the Foxa2-binding site is the negative regulatory element in the *FTO* promoter.

### Foxa2 down-regulates the Expression of *FTO* Gene

**Figure 4:**
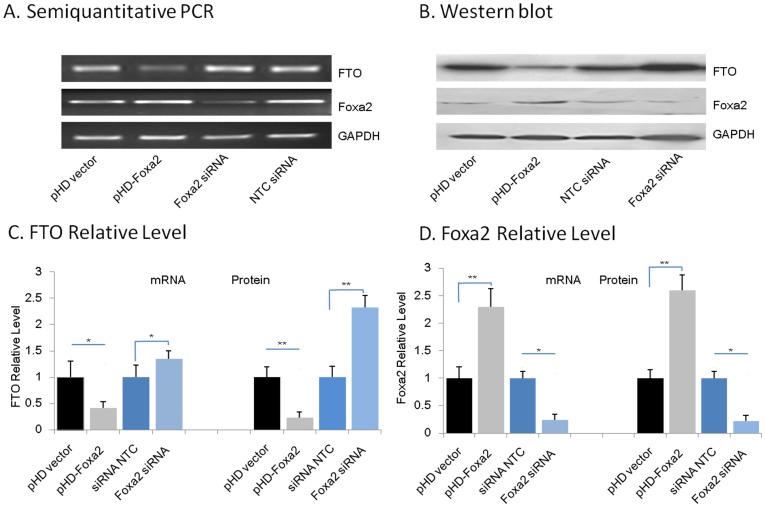
Foxa2 regulates the expression of FTO in HEK 293 cells. HEK 293 cells were transiently transfected with either a control vector (pHD vector) or a vector containing full coding sequence of *Foxa2* (pHD-Foxa2). In the RNA interference group, cells were transfected with siRNA against Foxa2 (Foxa2 siRNA) or negative control siRNA (NTC). After 48 h, cells were harvested and FTO expression was determined by qPCR and Western analyses. Bar graph depicts the relative mRNA and protein expression levels of FTO and Foxa2 as quantified by the Image Lab software (BIO-RAD) and normalized to GAPDH. Values represent the mean±SE of 3 independent experiments. (*P<0.05, **P<0.01).

To further explore the role of Foxa2 in regulating *FTO* expression level, a Foxa2 expression vector or a Foxa2 siRNA was transfected into HEK 293 cells. *FTO* mRNA and protein levels were detected by qRT-PCR or Western blotting, respectively. As illustrated in Fig.4, overexpression of Foxa2 resulted in a reduction of *FTO* transcript level by 60% and FTO protein level by 58.4%. In contrast, RNAi-mediated knockdown of Foxa2 resulted in a 1.5-fold more *FTO* mRNA level than the negative control siRNA and a concomitant increase of 45.5% in the FTO protein level.

### Foxa2 Binds to the *FTO* Promoter *in vitro*

**Figure 5:**
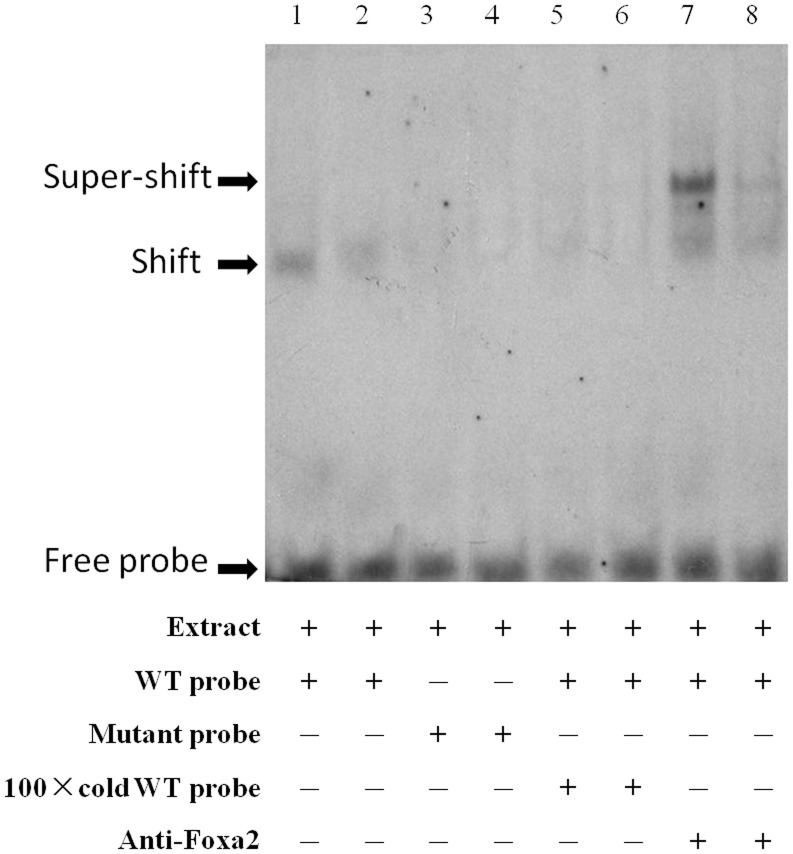
EMSA assays showing direct binding of Foxa2 to the *FTO* promoter *in vitro*. The EMSA assay showing direct binding of nuclear proteins to *FTO* promoter sequence using nuclear extract from HEK 293 cells (lane 1, 2). The competition assay was carried out by adding mutanted probes (lane 3, 4) or 100×unlabeled double-stranded oligonucleotide probes (lane 5, 6). The super-shift assay conducted using 20 ng anti-Foxa2 antibody (lane 7, 8). Each group was replicated with two lanes. The arrows indicate the probes, DNA-protein complexes and shift band.

As a transcriptional regulator, Foxa2 is essential for regulating of target gene expression via direct binding to the promoter. We employed EMSA to determine whether Foxa2 protein binds to the promoter of *FTO* gene *in vitro*. A biotin-labeled 20-nt fragment (−30 nt to −11 nt) containing the Foxa2 binding sites of the *FTO* promoter used in this assay. In competitive binding assays, unlabeled nucleotide sequence (cold WT probe) and a mutant probe with 5 bp nucleotide substitutions in the motif was used as competitor. To figure out whether the binding of DNA-protein complex is specifical, super-EMSA experiments were undertaken using the anti-Foxa2 antibody. Competition studies using mutanted EMSA probes showed that the bands of DNA-protein complexes were vanished in lane 3 and 4. Additionally, these shifted bands could be eliminated by the 100-fold excess of cold probe (lane 5, 6). For the super-EMSA studies shown in the last two lanes, the complex was super shifted by antibody when incubated with nuclear extracts (Fig. 5). Taken together, our results suggest that Foxa2 specifically binds to the predicted motifs to regulate *FTO* transcription directly.

### Foxa2 Binds to the *FTO* Promoter *in vivo*

**Figure 6:**
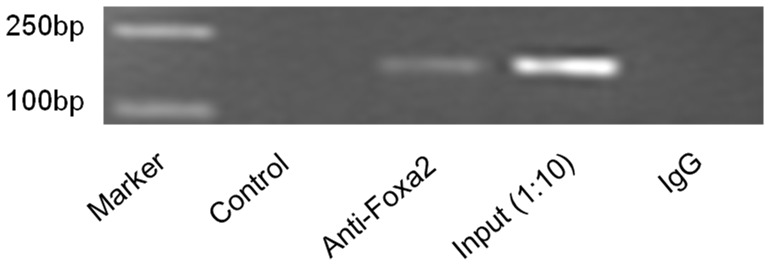
ChIP assays to test the binding of Foxa2 to the promoter of *FTO in vivo*. Formaldehyde cross-linked chromatin prepared from HEK 293 cells was immunoprecipitated with a Foxa2 antibody. PCR was performed with special primers to amplify a 156 bp DNA fragment containing Foxa2 binding sites. Sonicated decrosslinked DNA was used as positive input control for PCR. ChIP analysis reveals that Foxa2 could bind the sequence about 100 bp away from the transcription start site of *FTO*.

To further determine whether Foxa2 binds to the *FTO* promoter, EMSA and ChIP analysis was performed on HEK 293 nuclear samples. After coimmunoprecipitation using the indicated antibodies, DNA was extracted. Special PCR primers designed to amplify upstream and downstream of the Foxa2 binding sites were used to quantify the binding of Foxa2 to the *FTO* promoter. A negative control without antibody and a positive control with input DNA without IP (input) were included. As can be seen in Fig. 6, there was no signal detectable in the negative control amplicon. Conversely, a band of 156 bp was observed from the immunoprecipitated chromatin. These data demonstrate that the Foxa2 transcription factor is capable of binding to the *FTO* promoter region.

## Discussion

*FTO* was first discovered in a genome-wide association study (GWAS) for type 2 diabetes. In a number of studies conducted on multiple populations and different age groups, SNPs in the first intron of *FTO* were correlated with body weight and food intake [5]–[7], [10], [21]. However, *Fto−/−* mice show normal food intake but have an elevated metabolic rate [22]. In addition, studies have shown FTO to be expressed in several tissues, especially in specific parts of the brain affecting feeding regulation, such as the arcuate nucleus (ARC), the dorsal medial hypothalamus (DMH), and the ventral medial hypothalamus (VMH). Interestingly, the relationship of FTO expression and BMI is yet to be confirmed. Prior researches have focused on the relationship with mixed results. Brennan et al. demonstrated that FTO is associated with a risk for lung cancer using a Mendelian randomization approach [12]. The results from clinical pathology analysis showed that FTO is expressed in both normal and malignant breast tissue, and the SNPs are significantly associated with breast cancer risk [2]. In the present study, we cloned and analyzed the human *FTO* promoter to gain a better understanding of its transcriptional regulation.

To understand the regulatory mechanisms controlling *FTO* gene expression, we characterized the promoter region of *FTO*. Using 5′-deletion luciferase reporter constructs of the *FTO* promoter, we found the core promoter to be located in the −201 bp before the TSS. Bioinformatics analysis of the promoter sequence identified putative Foxa2 binding sites. Data from both overexpression and siRNA knockdown experiments suggest that Foxa2 is a negative regulatory element capable of inhibiting *FTO* promoter activity and expression level. EMSA and ChIP assays further demonstrate that Foxa2 binds to a conserved motif in the *FTO* promoter *in vitro* and *in vivo*.

Foxa2 is well known to be a critical transcription factor regulating glycogen and lipid metabolism. Recently, it was shown that the activation of Foxa2 is implicated in the regulation of several genes involved in liver and pancreas development [23]–[25]. Foxa2 has been shown to affect hepatic glycogen levels. Overexpression of Foxa2 in hepatocytes leads to a drop in glycogen levels. This is partly due to a reduction in glycogen synthase. Furthermore, Foxa2 induces the expression of pref-1, a known inhibitor of adipogenesis, consequently restraining preadipocyte differentiation. On the other hand, Foxa2 induces expression of Hsl, Glut-4, Hk-2, M2Pk, Ucp-2, and Ucp-3 in mature 3T3-L1 adipocytes. It has been suggested that the Foxa2 expression level in adipocytes could influence the development and progression of obesity. In *in vitro* differentiated primary human preadipocytes and in Simpson-Golabi-Behmel syndrome (SGBS) preadipocytes, *FTO* expression is downregulated during adipogenic differentiation. These findings are consistent with our results, in that *FTO* is a target gene of Foxa2.

Furthermore, Foxa2 binds to melanin-concentrating hormone (MCH) and orexin promoters and stimulates their expression [19]. MCH and orexin are neuropeptides expressed in the lateral hypothalamic area to regulate appetite and food intake in fasted mice. Foxa2 mutant mice exhibit increased fat deposits through impaired metabolism, with profound changes in insulin-mediated glucose uptake and metabolism. These physiological changes correlate with reductions in the expression of genes involved in glucose uptake and metabolism. Interestingly, we observed Foxa2 to bind to the *FTO* promoter and repress its transcription. Homeostasis within the organism is maintained through cross-talk among signaling networks. Indeed, the balance of food intake and other biological actions rely on such mechanism. Subtle regulation in the expression levels and balance among the genes in the “feeding center” would affect = food consumption, metabolism and insulin sensitivity. The siRNA-mediated functional knockdown of Foxa2 increased the transcriptional activity of *FTO*, which makes Foxa2 an interesting target in the therapy to improve the energy metabolism of overweight and obese people.

There is only one seminal study so far on the transcriptional regulation of *FTO*. Stratigopoulos. G et al. discovered that the SNP rs8050136 within the *FTO* intron affects the binding of isoforms of transcription factor Cut-like Homeobox 1 (CUX1) with its regulatory site, resulting in the altered expression level of *FTO* [26]. This is not inconsistent with our results because the transcription of a gene is dependent on several factors including enhancer, silencer, cofactors, and post-translational activators. In fact, SNPs influence transcriptional regulation in part by modifying the binding sites for transcription factors.

In conclusion, the core promoter region involved in the regulation of the human *FTO* gene promoter is located within the −200 bp upstream of the TSS. We presented the first data demonstrating that Foxa2 is a key regulator of the human *FTO* gene; it down-regulates the promoter activity and expression of *FTO*. It is likely that Foxa2 may be involved in the regulation of diet intake and metabolic functions. FTO can act as a crucial factor in accommodating metabolic signals, adapting behavior and physiological responses.
